# Effects of synbiotics preparations added to Pengging duck diets on egg production and egg quality and hematological traits

**DOI:** 10.14202/vetworld.2022.878-884

**Published:** 2022-04-10

**Authors:** Sri Kismiati, Luthfi Djauhari, Dwi Sunarti, Teysar Adi Sarjana

**Affiliations:** Department of Animal Science, Faculty of Animal and Agricultural Sciences Diponegoro University, Tembalang Campus, Semarang, Central Java, Indonesia

**Keywords:** cholesterol, egg production, egg quality, inulin, late phase, Pengging duck

## Abstract

**Background and Aim:**

Duck eggs have high cholesterol levels; inulin addition combined with probiotic is known in several studies to lower cholesterol, while maintaining egg production capacity and blood hematology. This study aimed to investigate the effect of the addition of synbiotic preparations on egg production, egg quality, and hematology of Pengging ducks.

**Materials and Methods:**

A total of 200 female Pengging ducks aged 75 weeks (late production phase) and weighing 1467±90.87 g were maintained in litter cages, each measuring 1×1 ducks. The treatment included the addition of synbiotics between the inulin of gembili tuber (*Dioscorea esculenta* L. and *Lactobacillus plantarum* Ina CC B76) as follows: T0=control feed (“farmer feed”), T1=control feed+synbiotics 1 mL/100 g, T2=control feed+synbiotics 1.5 mL/g, and T3=control feed+synbiotics 2 mL/100 g in the feed. A completely randomized design was used in this study. The production performance, physical and chemical qualities of eggs, and hematological parameters of Pengging ducks were evaluated.

**Results:**

The addition of synbiotics had no significant impact on the production performance, physical and chemical qualities of eggs, and hematological parameters (p>0.05), except for the egg yolk cholesterol content. The cholesterol content decreased significantly (p<0.05) with T2 and T3 treatments, but they had no significant effect (p>0.05). A significant decrease (p<0.01) in cholesterol levels was observed when the synbiotic dose was given at 1.5 ml/100 g feed (T2). However, there was no further decrease in cholesterol level when the synbiotic dose was increased to 2 ml/100g fed (T3)

**Conclusion:**

The addition of synbiotics preparations at 1.5 mL/100 g reduced the cholesterol content but did not improve egg production, egg physical quality, and hematology of Pengging ducks.

## Introduction

The Pengging duck is an Indonesian local egg producer duck. The first egg-laying age is approximately 6 months, with an egg production rate of 110-130 eggs/year [1]. In general, duck maintenance is still performed using a low-quality ration (“farmer feed”), due to which egg production and egg physical quality are very low, especially in the late production phase. Akruyek and Okure [2] mentioned that after the age of 50 weeks, egg production decreases, and egg weight increases; however, the weight and thickness of the eggshell, the pH of albumen and yolk, yolk index, and albumen index decrease. Duck eggs contain complete nutrition, that is, protein 10.7% [3], omega-3 and omega-6 [4], cholesterol 2.04 mg/g, and fat 31.88% [5]. Some consumers have an issue with the high content of cholesterol in duck eggs. It has been reported that the consumption of a half egg per day on a regular basis could increase the risk of developing cardiovascular disease in adults [6].

Feed additives are widely used in diets to improve the production and quality of chicken eggs, whereas in the case of duck eggs, the same is still extremely limited. Kiczorowska *et al*. [7] reported that feed additives increase the production performance of monogastric animals. Moreover, studies have shown that synbiotics supplementation (a combination of prebiotics and probiotics) as a feed additive improves health, nutrient absorption, and livestock production performance [8]; increases hemoglobin concentration; decreases the heterophil-to-lymphocyte (H/L) ratio [9]; increases eggshell weight and thickness [10]; and increases the hen-day egg production at 19, 20, 21, 22, and 23 weeks of age [11]. Furthermore, the addition of synbiotics could also decrease egg cholesterol levels [12]. The administration of a probiotic (*Saccharomyces* spp. Kb-5) was found to decrease the cholesterol content of yolk and increase the egg mass, feed efficiency, feed digestibility, yolk color, yolk and eggshell weight, shell thickness, and Ca content in the eggshell and yolk of duck eggs [13]. Natural feed additives consisting of natural ingredients, probiotics, and phytobiotics were found to increase egg production and egg quality and decrease the cholesterol content of Mojosari duck egg yolk [14]. Furthermore, Savedboworn *et al*. [15] reported that the use of inulin as a prebiotic increased the viability of *Lactobacillus plantarum*. Synbiotics (a mixture of inulin and *L. plantarum*) evidently inhibited the proliferation of pathogenic bacteria [16] and improved intestinal morphology, metabolizable energy, and nitrogen retention [17]. The improvement of intestinal morphology increases nutrient absorption, thereby improving physical quality and reducing the egg cholesterol content [18]. Shehata *et al*. [19] reported that synbiotics could reduce cholesterol content through the mechanisms of bile deconjugation with bile salt hydrolase (BSH), binding of cholesterol to the cellular surface of cells, coprecipitation of cholesterol with deconjugated bile, and incorporation of cholesterol into cellular membranes and short-chain fatty acids. They found that conjugated bile salts were regularly recirculated back into the enterohepatic circulation, whereas the circulating deconjugated bile salts were less soluble and eliminated in the excreta.

Gembili (*Dioscorea esculenta*) tuber contains 14.77% inulin [20] and bioactive compounds of water-soluble polysaccharides, dioscorin, and diosgenin [21]. The oligosaccharide of gembili tuber contains 0.231% lactulose, 2.541% inulin, and 1.485% raffinose [22]. Crespo *et al*. [23] mentioned that the inulin content of gembili tuber was 161 mg/100 g, which is higher than that of dahlia (*Dahlia pinnata*) tuber, and may potentially serve as a prebiotic.

In the present study, inulin derived from gembili was combined with *L. plantarum* Ina CC B76. Although synbiotics (based on inulin and gembili tubers and *L. plantarum*) have been used in broilers [17,24,25], to the best of the authors’ knowledge, they have never been used in ducks, especially in the late production phase.

This study aimed to evaluate the effect of the addition of synbiotics (inulin of gembili tuber and *L. plantarum* InaCC B76) to “farmer feed” on the production, physical and chemical qualities, especially cholesterol content, and hematology of Pengging duck eggs in the late production phase.

## Materials and Methods

### Ethical approval

The procedure of using duck in this study has been approved by the Animal Ethics Committee in the Faculty of Animal Sciences, Diponegoro University, Semarang, Indonesia, approval number 57-09/A-6/KEP-FPP.

### Study period and location

This research was conducted from September to December 2021. Samples were collected from Duck Breeding and Rearing Unit “Satker Itik Banyubiru”, Semarang District, Central Java.

### Animals

In this study, 200 female Pengging ducks aged 75 weeks (late production phase) with uniform body weight (1467±90.87 g) were used; they were maintained in an open-sided housing system. During the maintenance period, temperature and relative humidity were 21.64-29.86°C and 60.39-88.36%, respectively. Basal diet was formulated based on the “farmer feed” with 14.23% protein content, fat 4.58%, crude fiber 14.41%, and 2,403.74 kcal/kg EM and synbiotics (inulin of gembili tuber and *L. plantarum*) with a total bacterial count of 5.8×10^8^. The “farmer feed” consisted of 32.5% yellow corn, 40% rice bran, and 27.5% commercial concentrate, a product of PT Charoen Pokphand Indonesia Tbk (Hi-Pro-Vite 144). The nutrient content of Hi-Pro-Vite 144 concentrate is protein 37.0%-39.0%, fat 2%, crude fiber 6%, Ca 12%, P 1.2%, and 1750-1850 kcal/kg EM. The feed ingredients and nutritional content of “farmer feed” are presented in Table-1.

**Table 1:** Composition of feed ingredient and nutritional content of “farmer feed”.

Feed ingredient	Percentage
Yellow corn	32.50
Rice bran	40.00
Concentrate^1^	27.50
Nutrient content (%)	
Metabolizable energy (kcal/kg)^2^	2403.74
Crude protein (%)^3^	14.23
Crude fat (%)^3^	4.08
Crude fiber (%)^3^	14.41

1 Product of PT Charoen Pokphand Indonesia Tbk (Hi-Pro-Vite 144),

2 Metabolizable energy was calculated according to formula of Bolton cited by Sugiharto *et al*.^[52]^ as follows 40.81 {0.87 [crude protein+2.25 crude fat+nitrogen-free extract]+2.5},

3 Proximate analysis of Laboratorium Nutrition and Feed Science, Faculty of Animal and Agricultural Sciences, Diponegoro University

### Experimental design

A completely randomized design, with four treatments and five repetitions, was used in this study. Treatments consisted of the addition of synbiotics (inulin of gembili tuber and *L. plantarum*), namely, T0=control feed (without synbiotics), T1=control feed+synbiotics 1 mL/100 g ration, T2=control feed+synbiotics 1.5 mL/100 g ration, and T3=control feed+synbiotics 2 mL/100 g ration.

### Preparation of synbiotics

Gembili was harvested at the age of approximately 9 months and obtained from Pati, Central Java, and *L. plantarum* InaCC B76 was a product from the Indonesian Institute of Sciences. Gembili was washed, peeled, sliced, sun-dried, and then mashed (gembili tuber flour). Inulin was extracted using the method developed by Setyaningrum *et al*. [25]. Briefly, gembili tuber flour was added to hot water (90°C) in a ratio of 1:15, heated in a water bath at 80°C for 1 h, and then filtered using a filter cloth. The resulting filtrate was precipitated with 40% ethanol and stored in a freezer for 6 h. It was then removed from the freezer, allowed to melt, and then centrifuged at 3 075 x g for 5 min to obtain inulin deposits. The resulting precipitate was dried in an oven at 50°C and ground into inulin flour. Synbiotics were prepared by mixing 7 g/100 mL distilled water and 10 mL *L. plantarum* with a bacterial concentration of 1×10^9^ Colony-forming unit/mL and incubated at 37°C for 24 h.

### Diet preparation

The synbiotics were mixed with the feed by adding water before administration. The proportions of synbiotics, water, and feed are as follows: T1=5 mL:100 mL:500 g, T2=7.5 mL:100 mL:500 g, and T3=10 mL:100 mL:500 g.

### Tests and procedures

Egg production data were collected every day for 4 weeks, and feed consumption, egg weight, and egg mass were measured every day. Feed conversion was calculated by dividing feed consumption/individual/day by egg mass.

Data concerning egg physical quality (eggshell weight, eggshell thickness, eggshell strength, yolk weight, albumen weight, Haugh unit (HU), yolk index, yolk color, and albumen pH) were obtained by sampling that was conducted weekly, and the chemical quality of the yolk was measured by sampling three eggs/experimental unit in the final week of the study (78 weeks of age). The protein content was analyzed using the Kjeldahl method [26]. The fat content was measured by Soxhlet extraction. The calcium content was analyzed using an atomic absorption spectrophotometer (AAS-AA 6200 Shimadzu, Japan), and the yolk cholesterol content was determined using the enzymatic colorimetric method. First, the sample was saponified using methanolic KOH and then added with Fluitest kit cholesterol. Results were read using a spectrophotometer at 500 mm.

The analyses of cholesterol and triglyceride contents were conducted based on the cholesterol p-aminophenazone method [13], HDL and LDL analysis was based on the enzymatic colorimetric method [13].

Blood hematology was evaluated using a hematology analyzer at the end of the study using a sample of one duck per experimental unit. Approximately 2 mL blood was collected from the brachial vein and mixed with ethylenediaminetetraacetic acid, after which the hematological profile was analyzed. Hemoglobin level was measured by cyanide-free hemoglobin spectrophotometry, and erythrocyte, hematocrit, and leukocyte levels were determined using the electrical impedance method.

## Statistical analysis

Data were analyzed in a completely randomized design using one-way analysis of variance and Duncan’s multiple range test at the 5% significance level. Analysis was performed using Statistical Analysis System (SAS) for University Edition (https://www2.nau.edu/stat-lic/sas/sas-univ.html). Several data, such as hematological results, did not follow the normal distribution, and hence, the transformation was performed according to the characteristics of the data before the analysis of variance.

## Results

### Production performance

Table-2 shows the results of the effect of adding synbiotics (inulin extract of gembili tuber and *L. plantarum*) on the egg production and egg quality of Pengging ducks aged 78-82 weeks. The addition of synbiotics had no significant effect on feed consumption, egg production, egg weight, egg mass, and feed conversion of Pengging ducks.

**Table 2:** Production performance of Pengging duck of 75-78 weeks old when added with synbiotic (inulin of gembili tuber and *Lactobacillus plantarum).*

Parameter	Treatment				p-value	Standard error

	T0	T1	T2	T3		
Feed consumption (g)	148.66	149.37	148.73	149.05	0.79	0.53
Egg production (%/day)	20.43	18.43	24.57	25.43	0.76	0.52
Egg weight (g)	64.10	60.74	61.79	62.58	0.44	1.46
Egg mass	12.76	12.15	12.35	12.45	0.51	0.28
Feed conversion	9.50	10.45	9.45	10.83	0.16	0.49

### Physical and chemical egg quality

The addition of synbiotics had no significant impact on egg physical quality and yolk protein, fat, and Ca contents (p>0.05), but it had a significant (p<0.05) impact on yolk cholesterol (Table-3). The yolk cholesterol content decreased with the addition of 1.5 mL/100 g (T2) and 2.0 mL/100 g (T3) synbiotics to the feed.

**Table 3:** The physical and chemical egg quality of Pengging duck which given symbiotic (inulin of gembili tuber and *Lactobacillus plantarum)*.

Parameter	Treatment				p-value	Standard error

	T0	T1	T2	T3		
Eggshell weight (g/%)	6.11/9.5	5.98/9.5	5.95/9.5	5.62/8.9	0.38	0.04
Eggshell thickness (mm)	0.43	0.40	0.40	0.37	0.31	0.05
Eggshell strength	4.65	4.62	4.67	4.55	0.36	0.06
Yolk weight (g)	20.09/31.3	18.53/30.5	20.508/33.1	20.75/33.1	0.16	0.03
Yolk weight (%)	31.3	30.5	33.1	20.75/33.1	0.17	0.04
Albumen weight (g)	37.90/59.12	36.22/59.63	35.33/57.18	36.21/57.85	0.35	0.02
Albumen weight (%)	59.12	59.63	57.18	57.85	0.37	0.03
Haugh unit/HU	83.10	86.25	84.92	86.02	0.9	1.00
Yolk index	0.43	0.42	0.45	0.47	0.11	0.21
Yolk color	11.75	12.00	12.25	12.45	0.10	0.19
Albumen pH	7.80	7.84	7.90	7.94	0.37	0.05
Protein (%)	9.38	9.46	9.54	9.91	0.31	0.29
Fat (%)	17.85	18.52	17.85	17.64	0.08	0.23
Ca (%)	0.05	0.07	0.02	0.03	0.40	0.02
Cholesterol	2.23^b^	2.25^b^	0.57^a^	0.56^a^	<0.01	0.19

^a,b^ Means in the same row with different letters show significant differences (p<0.05)

### Hematology

As shown in Table-4, the addition of synbiotics had no significant effect on the hematological parameters of Pengging ducks (p>0.05).

**Table 4:** Hematology of Pengging duck which given symbiotic (inulin of gembili tuber and *Lactobacillus plantarum*).

Parameter	Treatment				p-value	Standard error

	T1	T2	T3	T4		
Leukocyte (×10^9^/L)	46.40	42.40	67.50	60.88	0.476	11.93
Erythrocyte (×10^12^/L)	2.61	2.85	4.82	7.39	0.509	1.68
Hemoglobin (g/dL)	12.60	12.20	13.10	10.38	0.811	1.69
Hematocrit (%)	30.10	28.30	25.30	26.00	0.899	4.41
Thrombocyte (×10^9^/L)	13.00	9.60	9.20	9.00	0.079	1.03
Lymphocyte (×10^9^/L)	44.24	50.54	63.50	56.63	0.677	10.25
Neutrophil (×10^9^/L)	6.10	7.90	3.60	14.38	0.596	4.65
Heterophil-to-lymphocyte ratio	0.26	0.16	0.07	0.24	0.778	0.11

## Discussion

### Production performance

Table-2 shows that the production performance was lower than in the previous research reports. Thus, Purwati *et al*. [27] reported that the average weight of Pengging duck egg was 63.66 g at a feed consumption of 160 g. The ducks used in their study were old or pre-molting; for this reason, their production performance was low. In old age, the ovarium and oviduct weight decreases [28], the levels of hormones required for the process of egg formation decrease, and egg production becomes low [29].

In the present study, 78-82-week-old ducks were used; thus, the addition of synbiotics (inulin of gembili tuber and *L. plantarum*) had no significant effect on feed consumption, feed conversion, egg production, egg weight, and egg mass (p>0.05). The ducks were in the late production phase/pre-molting phase; hence, the added synbiotics caused no changes in the intestinal villi and did not increase the nutrient digestibility and production performance. Along with the increasing age of ducks, there was a decrease in the levels of hormones required for egg formation, so egg production decreases, and the addition of the synbiotics could not increase egg production. According to Dibner and Richards [30], the structure, dynamics, and function of digestive organs are influenced by age and diminish with age. Older poultry had lower egg production [31]. Purbarani *et al*. [32] reported that feed containing 18% protein/low protein and fortified by a combination of 1.2% inulin of dahlia tuber and 1.2 mL *Lactobacillus* spp. increased the height of jejunum villi and growth of 8- to 70-day-old chicken. The combination of probiotics and phytobiotics in “standard feed” (17% protein and 2654 EM) was found to improve the histomorphology of the ileum. However, it did not significantly affect protein digestibility, feed conversion, and egg mass of laying hens aged 72-77 weeks [33]. Tang *et al*. [34] reported that the supplementation of synbiotics effectively increased feed consumption, feed conversion, egg production, egg weight, and egg mass of laying hens aged 20-36 weeks but had no significant effect in laying hens aged 37-52 weeks. The addition of 1.3% inulin to the feed increased the length of the small and large intestines, egg production, and egg weight of laying hens aged 57-60 weeks [35]. Consistently, Zarei *et al*. [10] showed that the use of chemical/commercial synbiotics did not significantly affect feed consumption, egg production, egg mass, egg weight, and feed conversion. However, this finding was different from that reported by Getachew [36], who showed that probiotic supplementation may increase egg production.

### Physical and chemical eggs quality

As shown in Table-3, the physical quality of eggs was not significantly different (p>0.05). This result confirmed that the addition of synbiotics (inulin of gembili tuber and *L. plantarum*) could not improve the egg physical quality of late-phase ducks. The supplementation of synbiotics to old ducks fed with low-protein diet (“farmer feed”) also did not increase egg quality. This result indicated that this synbiotic preparation cannot improve intestinal histomorphology, and the levels of hormones required for the process of egg formation in old ducks were low.

According to Table-3, the yolk egg, albumen, and eggshell weights were lower than previous research studies; Sun *et al*. [3] showed that the yolk weight of 50-week-old ducks was 24.06 g, whereas the egg white/albumen weight was 42.79 g. The eggshell weight of Pengging duck was 8.40 g, and the eggshell thickness was 0.29 mm [37]. Yolk color was within a normal range (11.75-12.45). According to Du *et al*. [38], the yolk color score of Shan Partridge ducks was 12.38, whereas the eggshell strength was 4.34 (kg.f), HU was 73.64, yolk weight was 24.56 g, and eggshell thickness was 0.43 mm. Yolk protein content was within the normal range. The protein content of duck was 9.24%, cholesterol content was 11.38%, egg white relative weight was 50.87%, and yolk relative weight was 32.68% [39].

The protein, fat, and calcium contents of yolk were not significantly different, but the cholesterol content was p<0.05. The addition of 1-2 mL/g synbiotics did not increase the egg protein and Ca contents, but at the levels of 1.5 and 2 mL/g, the yolk cholesterol content was reduced. In the present study, the “farmer feed” (very low protein) and very high crude fiber content (Table-1) were used so that despite synbiotics supplementation, the protein and Ca deposition in eggs were not significantly different (p<0.05). This result is consistent with the study of Sari *et al*. [24], who showed that supplementation with 0.5-1.5% of synbiotics into drinking water did not significantly affect the yolk protein and fat content of eggs.

In the present study on Pengging ducks aged 78-82 weeks and fed with low-nutrient feed/”farmer feed,” it is expected that the egg protein and Ca contents would increase and fat and cholesterol contents would decrease. However, the yolk protein, fat, and Ca contents were not significantly different, which may be because of the duck’s age. At this age, their intestinal morphology did not change. Thus, the intestine absorbed nutrients only adequately for body maintenance, and egg production (Table-2) and yolk protein and Ca contents did not increase (Table-3). Villagrán-de la Mora *et al*. [40] reported that supplementation of synbiotics in drinking water increased the number of lactic acid bacteria, villi length, and crypt depth and resulted in a better villous-to-crypt ratio. Synbiotics addition increased the height of villi in the duodenum and ileum of 35-week-old [41] and 48-week-old broilers[42]. However, supplementation with feed additives had no significant effect on the intestinal morphology of 73-week-old layers [43]. This was because the ducks were old, so the synbiotics did not increase the length of the villi, the depth of the crypts, and the ratio of the villi to crypts, and hence, nutrient absorption was not optimal. Prakatur *et al*. [44] reported that nutrient absorption was influenced by villus height and villus height-to-crypt depth ratio; that is, the greater the villus height and the villus height-to-crypt depth ratio, the higher the nutrient absorption.

As shown in Table-3, the addition of synbiotics (inulin of gembili tuber and *L. plantarum* 1.5 mL/100 g [T2]) was able to reduce the yolk cholesterol content (p<0.05). According to Getachew [36], supplementation with probiotics reduced chicken egg cholesterol content. Shehata *et al*. [19] mentioned that synbiotics could reduce cholesterol content through BSH in the enterohepatic circulation, which was then eliminated through excreta. The BSH is known to facilitate bile salt deconjugation.

The hypocholesterolemia effect of synbiotics was due to reduced cholesterol absorption from the gastrointestinal tract and/or by the deconjugation of bile salts in the intestine, which would prevent their reabsorption through the enterohepatic circulation. In a previous study, Elkin *et al*. [45] hypothesized that some caution is necessary regarding the cholesterol-lowering ability of probiotics or prebiotics because in most studies, the yolk weight was not reported. Concerning the hypocholesterolemia ability in the present study, it was manifested without affecting the HDP, the size or weight of the yolk, and the whole egg weight. Increasing cecum influx of polysaccharides could influence increasing microbial population [46]. Inulin is rich in complex polysaccharide compounds that may provide and contribute supporting substances for gut microbial proliferation, including lactic acid bacteria and other beneficial microorganisms. Similarly, it could cause alterations in the physical conditions of the digestive tract environment, such as an optimum intestine pH [47]. The availability of polysaccharides and an increased number of beneficial microorganisms can reduce blood cholesterol profiles [46], where a decrease in blood cholesterol levels was positively related to a decrease in egg cholesterol levels. The cholesterol-lowering effect of the probiotic was due to reduced cholesterol absorption from the gastrointestinal tract and/or by the deconjugation of bile salts in the intestine, which would prevent their reabsorption through the enterohepatic circulation [45].

### Hematology

There were no significant changes in the hematological status (p>0.05), as shown in Table-4. The addition of synbiotics successfully reduced the yolk egg cholesterol content without affecting the hematological status of the ducks.

This result was similar to that of the previous studies, where the addition of synbiotics to the feed was found to have no significant impact on the hemoglobin levels in the starter, grower, and finisher phases of broilers [47,48] and H/L of laying hens [10,49]. Similarly, Zbikowski *et al*. [50] and Tarabees *et al*. [51] reported that the use of synbiotics had no significant effect on the hematology of broilers.

## Conclusion

The addition of 1.5 mL/g synbiotics (inulin of gembili tuber and *L. plantarum*) preparations to the feed reduced the cholesterol content in the egg yolks of Pengging ducks. However, there was no improvement in egg production, egg physical quality, and hematology. The limitation of this study was conducted using Pengging ducks (Indonesian local duck), the egg cholesterol deposition response might be different in other types of ducks. In the future, the results can be implemented to produce duck eggs with lower cholesterol levels.

## Authors’ Contributions

SK: Conducted the research, data collection, and drafted the manuscript. DS: Developed the feeding concept and supervised the study. LD: Designed the experiment. TAS: Conducted data analysis. All authors read and approved the final manuscript.
